# Exosomal miR-155-5p drives widespread macrophage M1 polarization in hypervirulent *Klebsiella pneumoniae*-induced acute lung injury via the MSK1/p38-MAPK axis

**DOI:** 10.1186/s11658-023-00505-1

**Published:** 2023-11-13

**Authors:** Yihan Xu, Chunying Zhang, Danni Cai, Rongping Zhu, Yingping Cao

**Affiliations:** 1 Department of Clinical Laboratory, Fujian Medical University Union Hospital, Fuzhou, 350001 People’s Republic of China; 2 Central Laboratory, Fujian Medical University Union Hospital, Fuzhou, 350001 People’s Republic of China

**Keywords:** Hypervirulent *Klebsiella pneumoniae*, Acute lung injury, Macrophage, Exosome, miR-155-5p

## Abstract

**Background:**

Hypervirulent *Klebsiella pneumoniae* (hvKp) infection-induced sepsis-associated acute lung injury (ALI) has emerged as a significant clinical challenge. Increasing evidence suggests that activated inflammatory macrophages contribute to tissue damage in sepsis. However, the underlying causes of widespread macrophage activation remain unclear.

**Methods:**

BALB/c mice were intravenously injected with inactivated hvKp (iHvKp) to observe lung tissue damage, inflammation, and M1 macrophage polarization. In vitro, activated RAW264.7 macrophage-derived exosomes (iHvKp-exo) were isolated and their role in ALI formation was investigated. RT-PCR was conducted to identify changes in exosomal miRNA. Bioinformatics analysis and dual-luciferase reporter assays were performed to validate MSK1 as a direct target of miR-155-5p. Further in vivo and in vitro experiments were conducted to explore the specific mechanisms involved.

**Results:**

iHvKp successfully induced ALI in vivo and upregulated the expression of miR-155-5p. In vivo, injection of iHvKp-exo induced inflammatory tissue damage and macrophage M1 polarization. In vitro, iHvKp-exo was found to promote macrophage inflammatory response and M1 polarization through the activation of the p38-MAPK pathway. RT-PCR revealed exposure time-dependent increased levels of miR-155-5p in iHvKp-exo. Dual-luciferase reporter assays confirmed the functional role of miR-155-5p in mediating iHvKp-exo effects by targeting MSK1. Additionally, inhibition of miR-155-5p reduced M1 polarization of lung macrophages in vivo, resulting in decreased lung injury and inflammation induced by iHvKp-exo or iHvKp.

**Conclusions:**

The aforementioned results indicate that exosomal miR-155-5p drives widespread macrophage inflammation and M1 polarization in hvKp-induced ALI through the MSK1/p38-MAPK Axis.

**Graphical Abstract:**

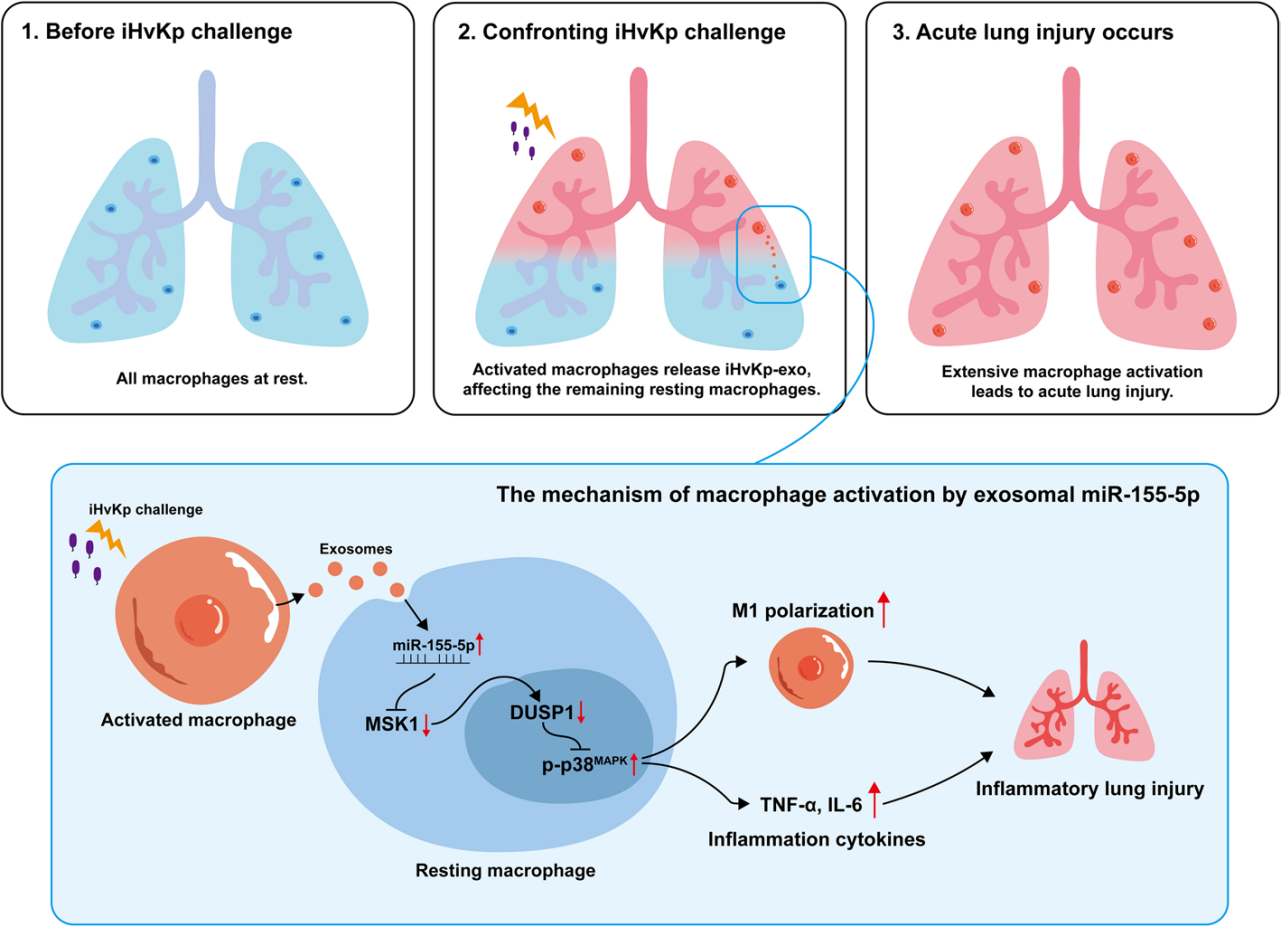

**Supplementary Information:**

The online version contains supplementary material available at 10.1186/s11658-023-00505-1.

## Background

Hypervirulent *Klebsiella pneumoniae* (hvKp) is a significant Gram-negative pathogen in hospital infections [1]. In recent years, the proportion of clinically isolated hvKp strains has been increasing annually [2]. HvKp is highly invasive and pathogenic, and it can cause severe sepsis or septic shock, often accompanied by a severe complication called acute lung injury (ALI) [3]. About sepsis, the main mechanism involves the induction of widespread and uncontrolled inflammatory response throughout the body by bacterial components, resulting in extensive interstitial and alveolar edema [4]. It has been reported that hvKp-induced sepsis-related ALI has a mortality rate of over 50% [5]. Therefore, the occurrence of highly hvKp infection-induced sepsis and related ALI has become an increasingly significant clinical challenge.

Macrophages play a crucial role in the development of lung inflammation and tissue damage in Gram-negative bacterial infections [6]. Macrophages can be polarized into M1 or M2 phenotypes [7]. During infection, macrophages polarize toward the M1 phenotype upon encountering foreign pathogens and secrete pro-inflammatory cytokines such as TNF-α, IL-1β, IL-6, and IL-12 [8, 9]. One of the causes of sepsis-related tissue damage is thought to be the extensive and excessive M1 polarization and inflammatory activation of macrophages [10]. Evidence suggests that in lipopolysaccharide (LPS)-induced ALI animal models, lung macrophages undergo extensive M1 polarization, which further exacerbated the tissue damage during sepsis [11]. Therefore, uncovering the mechanisms underlying the widespread activation of macrophages during sepsis may offer fresh perspectives on the clinical management of ALI.

Exosomes are extracellular vesicles secreted by cells, with a diameter ranging from 40 to 160 nm, and they contribute significantly to intercellular communication [12, 13]. Evidence suggests that exosomes derived from serum in septic mouse models can promote macrophage proliferation and inflammatory response [11]. Exosomes can carry various cargo, including microRNAs [14]. MicroRNAs are important non-coding small RNAs that regulate post-transcriptional translation by targeting and degrading specific mRNA molecules [15]. Previous studies have reported that miR-155-5p can regulate the inflammatory response by targeting SOCS1 [16]. After being absorbed by recipient cells, exosomes release their microRNA cargo into the cytoplasm, thereby modulating the functions of the target cells [17]. In sepsis, there have been studies on the pro-inflammatory activation of macrophages by exosomes and their miRNAs [11, 18, 19]. For instance, exosomes derived from neutrophils can induce polarization and pyroptosis in macrophages through the delivery of miR-30-5p [19]. Thus, exosomes hold potential as a novel therapeutic target for various diseases, such as acute inflammation. However, in the development of sepsis-related ALI, the role of exosomes derived from macrophage activation and their miRNAs remains unknown. Hence, we aim to elucidate the contribution of exosomes derived from activated macrophages and their cargo of miRNAs in the pathogenesis of ALI induced by hvKp.

In our study, we uncovered that exosomal miR-155-5p derived from macrophages activated by iHvKp can influence the M1 polarization and inflammatory response of resting macrophages by activating the p38-MAPK signaling pathway, thereby affecting lung inflammation and tissue damage. These findings provide a new explanation for the widespread activation of macrophages in ALI. Additionally, exosomal miR-155-5p and its novel target gene, mitogen- and stress-activated protein kinase-1 (MSK1), may serve as new targets for the clinical treatment of inflammatory tissue damage in pneumonia.

## Methods

### Animals

Adult BALB/C mice were obtained from SiPeiFu Biotechnology (Beijing, China). All animal research protocols were approved by the Animal Experiment Ethics Committee of Fujian Medical University (Issue No.: IACUC FJMU 2023-0062). All experiments conform to all relevant regulatory standards.

### Bacterial culture

HvKp strains were obtained from the laboratory’s collection. Bacteria were identified based on previously reported characteristics [20]. Bacteria were cultured in LB (Haibo, China) medium at 35 °C with shaking for 4–6 h. After that, the bacterial cultures were then centrifuged at 5000 *g* for 15 min to collect bacteria. Then add 10 mL PBS and resuspended pellet, followed by another centrifugation at 5000*g* for 15 min, and discard supernatant again. The pellet was resuspended in PBS to obtain a bacterial suspension. Bacterial concentrations were assessed by serial dilutions and quantified by measuring the optical density at 600 nm (OD600). The bacterial suspension was further diluted to achieve the desired colony-forming unit (CFU) concentrations for each experiment.

#### Induction of ALI mouse model with IHvKp

The suspension of hvKp was inactivated using autoclaving. Briefly, the bacterial suspension in glass vials was covered with autoclave indicator tape and subjected to a high-pressure autoclaving program at 121.3 °C for 15 min. If the indicator tape changed color after autoclaving, it indicated successful bacterial inactivation.

To establish the ALI mouse model, three male BALB/C mice were intravenously injected with iHvKp (10^10^ CFU/mouse), and an equal number of mice were injected with PBS as a negative control. At 24 h after injection or when humane endpoints were reached, such as decreased body temperature (lower than 30 °C), poor response to external stimuli, or inability to move freely, mice were euthanized and collected broncho-alveolar lavage fluid (BALF) and lung tissues.

### Lung tissue analysis

Lung tissues were fixed in 4% paraformaldehyde for 24 h. The fixed tissues were then embedded in paraffin, sectioned, and stained with hematoxylin and eosin (HE) for microscopic examination.

#### MPO assay

Myeloperoxidase (MPO) activity was measured to assess the accumulation of inflammatory cells. Lung tissues were homogenized in buffer (*w*/*v* = 1:19), and MPO activity was determined using the MPO-test kit (ZCIBIO, China).

#### BCA assay

The total protein concentration in the BALF was measured to assess lung permeability using a BCA assay kit (Vazyme, China), following the manufacturer's instructions.

#### Cell culture

RAW264.7 (Cat. CL-0190) or HEK293T (Cat. CL-0005) cells were obtained from Procell (Wuhan, China). Cells were cultured in a specialized culture medium (Procell, China). Exosomes and cells were co-cultured using an exosome-specific medium (Umibio, China).

RAW264.7 activation was achieved by co-culturing with iHvKp. The cells were treated with a multiplicity of infection (MOI) of 100:1 (bacteria to cells) in culture dishes. After 48 h, cells and supernatants were collected. Cell activation was assessed by determining M1 polarization and inflammatory response, and the supernatant was collected for exosome isolation. The negative control supernatant was obtained by replacing iHvKp with PBS during the stimulation process.

### Exosome isolation, characterization, labeling, and uptake

Exosomes were isolated using the exosome isolation and purification kit (Umibio, China), following the instructions provided. In brief, the collected cell culture supernatant was centrifuged at 3000*g*/min for 10 min to eliminate cells. After that, the supernatant was mixed with exosome concentration solution, vigorously shaken for 1 min, and incubated overnight at 4 °C. The mixture was then centrifuged at 10000*g*/min for 1 h, and the precipitate was retained and resuspended in PBS. Subsequently, another centrifugation at 12000*g*/min for 2 min was performed, and the resulting supernatant, passed through an exosome purification filter, yielded the exosome solution.

After isolation, exosomes were characterized using nanoparticle tracking analysis (NTA) and transmission electron microscopy (TEM). Additionally, protein markers such as ALIX, TSG101, and CD63 were identified through Western blot analysis.

To investigate whether exosomes were taken up by cells, they were labeled with PKH67/PKH26 before injection or co-culture. The labeling was performed using the exosome PKH67/PKH26-label Kit (Umibio, China), following the provided instructions. Briefly, exosome solution (200 μl) was mixed with the staining solution (50 μl), vigorously shaken for 1 min, and incubated for 10 min. After the incubation, the excess dye was removed by exosome extraction, and the resulting solution contained PKH67-labeled exosomes. After injection or co-culture for 6 h, lung tissues or cells were fixed, stained with DAPI, and observed under a fluorescence microscope.

### TEM

The exosome samples (20 μl) were carefully added drop by drop onto 200-mesh grids and incubated at room temperature for 10 min. Following this, the grids were subjected to negative staining using 2% phosphotungstic acid for 3 min. Excess liquid was removed using filter paper, and the samples were subsequently examined using an HT7800 transmission electron microscope (Hitachi, Japan).

### In vivo injection and in vitro co-culture of exosomes

Quantification of isolated exosomes was performed using the BCA assay as described previously [21]. In vivo, three male BALB/C mice per group were intravenously injected with PBS-exo or iHvKp-exo (300 μg/mouse), while an equal number of mice were injected with PBS as a negative control. In vitro, RAW264.7 cells were co-cultured with PBS-exo or iHvKp-exo (50 μg/ml).

### Nucleic acid transfection

Transfection of miR-155-5p inhibitor into mouse lung tissues was performed using in vivo-jetPEI (Polyplus, France). Eight male BALB/C mice per group were intravenously injected with 200 μl of a 5% glucose solution containing 6.4 reagent and 40 μg of miR-155-5p inhibitor or negative control one day before exosome or iHvKp injection.

For cell transfection of miR-155-5p inhibitor, miR-155-5p mimic, and their respective negative controls, the lipo3000 reagent kit (Invitrogen, USA) was used. The transfection mixture was prepared with a ratio of 10 pg of nucleic acid: 0.3 μl of lipo3000: 0.5 μl of P3000: 20 μl of MEM medium each well of a 48-well plate.

### Immunofluorescence staining

Immunofluorescence staining was performed as previously described [22]. In brief, lung sections were subjected to an overnight incubation with either a mouse CD68 antibody (macrophage marker, Cat.GB113109, Servicebio, China) or an iNOS/Arg1 antibody (macrophage polarization marker, Cat.GB11119/ GB11285, Servicebio, China) at 4 °C. Following thorough washing, Alexa Fluor 488-conjugated (Cat.GB25303) or CY3-conjugated (Cat.G1223) secondary antibodies (Servicebio, China) were applied. The nucleus was stained with DAPI. The sections were visualized using a DMi8 fluorescence microscope (Leica, Germany).

### Western blotting

Western blot analysis was performed as described previously [23]. Briefly, protein lysates of cells or exosomes were resolved on SDS-PAGE gels and then transferred onto polyvinylidene difluoride membranes for identification. GAPDH was used as an internal control. The following antibodies were used: anti-CD63 (1:500, Cat.ab216130, Abcam), anti-ALIX (1:2000, Cat.E6P9B, CST), anti-TSG101 (1:2000, Cat.ab125011, Abcam), anti-MSK1 (1:500, Cat.3489S, CST), anti-DUSP1 (1:1000, Cat.ab138265, Abcam), anti-p-p38^MAPK^ (1:1000, Cat.4511T, CST), anti-p38^MAPK^ (1:1000, Cat.8690S, CST), and anti-GAPDH (1:3000, Cat.92310SF, CST).

### Nucleic acid extraction and identification

Total RNA extraction from cells or exosomes was performed using the Trizol method as previously described [12]. The total RNA extracted from cells was reverse transcribed using a reverse transcription kit (Vazyme, China). Quantification of RNA was carried out using an RT-PCR kit (Quanshijin, China). Reverse transcription and quantification of exosomal miRNA were performed using a specific kit (Tiangen, China). Data were normalized to the expression of GAPDH or U6. All RT-PCR analyses were performed using an ABI 7500 instrument. The primers are listed in Additional file 1: Table S1.

### Dual-luciferase reporter assay

To investigate the interaction between miR-155-5p and the 3ʹ untranslated region (UTR) of the MSK1 gene, plasmid vectors containing the wild-type and mutant versions of the MSK1 3ʹ UTR with predicted miR-155-5p binding sites were constructed. These constructs were transfected into HEK293T cells. Additionally, a renilla luciferase vector was co-transfected in all transfections to monitor the efficiency of transfection. Luciferase activity was quantified as relative light units, with the average activity of the Photinus pyralis firefly luciferase normalized to the average activity of the renilla luciferase vector.

### Flow cytometry

Macrophages were resuspended in PBS after centrifugation for FACS analysis. Anti-CD86 (BioLegend, Cat.105006, USA) was used according to the manufacturer's instructions. Data were acquired using a C6 flow cytometer (BD, USA).

### Statistical analysis

Statistical analysis was performed using GraphPad Prism software (version 8.0) or SPSS (version 25). The data were tested for normal distribution using the Shapiro–Wilk test. Normally distributed data are presented as mean ± standard error of the mean (SEM). Two-group comparisons were analyzed using two-tailed Student's t-test. Multiple group comparisons were performed using one-way analysis of variance (ANOVA) followed by post hoc tests. Survival rates between groups were compared using the Log-rank test. A *p*-value of less than 0.05 was considered statistically significant.

## Results

### Expression of miR-155-5p increases in iHvKp-induced mouse model of ALI

**Fig. 1:**
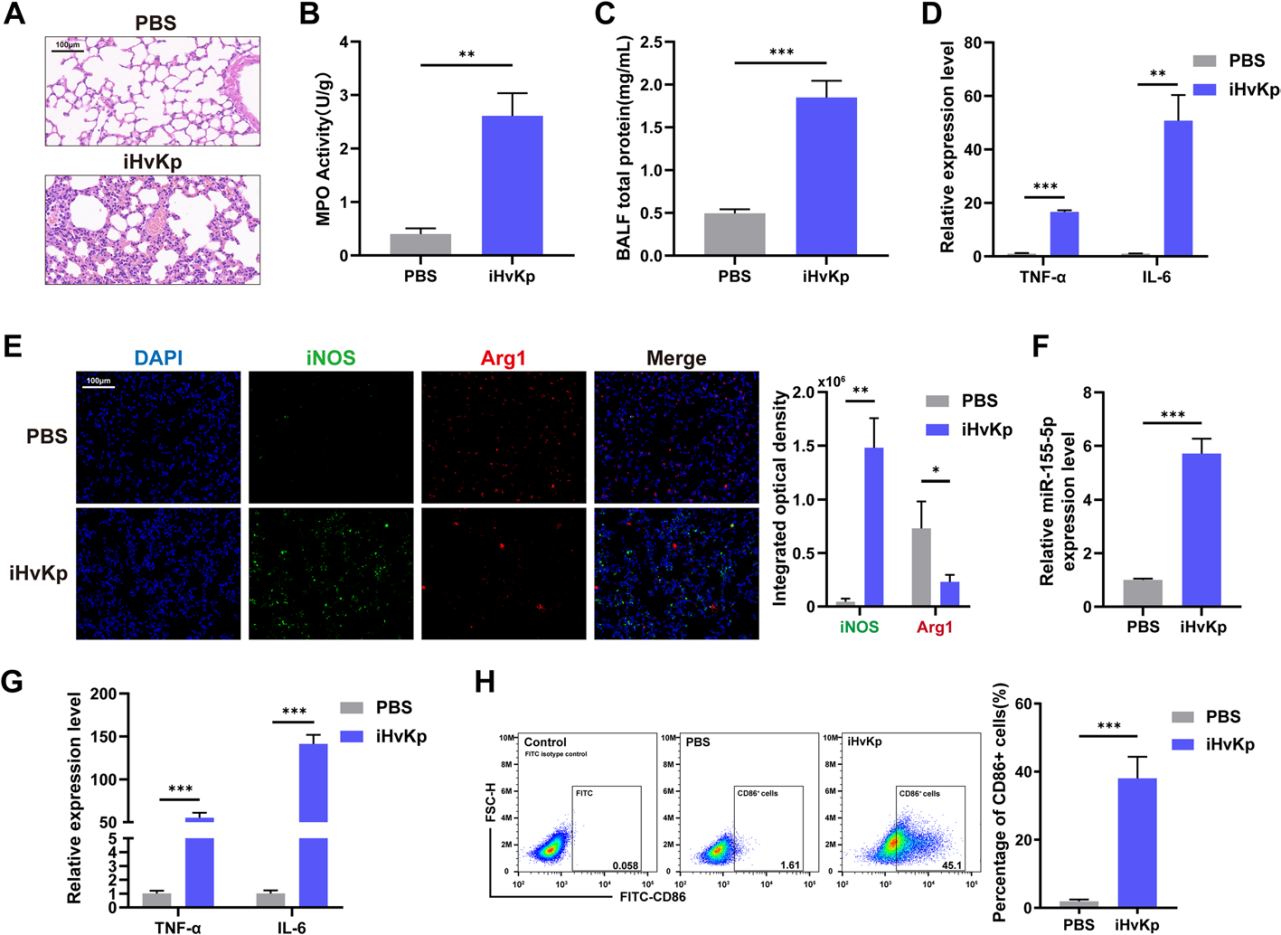
Expression of miR-155-5p increases in iHvKp-Induced mouse model of AL. **A** Lung tissue identification. The inflammatory response in lung tissues was determined by HE staining after iHvKp injection 12 h via the tail vein. **B** Recruitment of inflammatory cells assessed by MPO activity assay. **C** Lung permeability was assessed by measuring BALF total protein concentration using a BCA assay. **D** Levels of pro-inflammatory cytokines TNF-α and IL-6 measured by RT-PCR. **E** Immunofluorescence intensity of macrophage polarization cell markers in lung tissues, iNOS (green, M1) and Arg1 (red, M2). **F** Expression of miR-155-5p in lung tissues measured by RT-PCR. **G** Levels of pro-inflammatory cytokines TNF-α and IL-6 in iHvKp-stimulated RAW264.7 macrophages measured by RT-PCR. **H** Level of M1 polarization determined by flow cytometry. The data are presented as mean ± SEM, *p < 0.05, **p < 0.01, ***p < 0.001, *n* = 3

Sepsis is commonly reported to be induced by bacterial components [24]. To avoid interference from live bacteria in the ALI model, we used inactivated Hypervirulent *Klebsiella pneumoniae* (iHvKp) to establish a mouse model of ALI. This is the first report of iHvKp being used for ALI modeling, and some details of the model are shown in Additional file 2. To verify whether iHvKp can induce ALI in mice, we performed HE staining on lung tissues. The results showed significant interstitial edema, pulmonary congestion, and inflammatory cell infiltration in the lung tissues of iHvKp-challenged mice (Fig. 1A). MPO activity serves as a reliable indicator reflecting the accumulation of inflammatory cells. The results of the MPO activity assay indicated the presence of inflammatory cell infiltration in the lung tissues of iHvKp-challenged mice (Fig. 1B). The BALF total protein was used to assess lung permeability, and the results showed an increase in lung permeability in iHvKp-challenged mice (Fig. 1C). Furthermore, we detected the levels of two pro-inflammatory cytokines, TNF-α and IL-6, and observed an upregulation in their expression (Fig. 1D). These findings suggest that iHvKp induced ALI in mice.

Macrophage activation (specifically M1 polarization) has been widely acknowledged as a pivotal contributor to ALI pathogenesis in mice [25]. To further confirm whether macrophages are activated in iHvKp-induced ALI in mice, we examined the expression levels of iNOS (M1 marker) and Arg1 (M2 marker) in lung tissues. Immunofluorescence staining revealed a significant increase in iNOS and a decrease in Arg1 under iHvKp stimulation, indicated a widespread M1 polarization of macrophages in the mouse lung tissue. (Fig. 1E). These results indicate enhanced M1 polarization and inflammatory response of macrophages in iHvKp-induced mice. miR-155-5p has been reported to be associated with pulmonary inflammation [26], and our analysis also found an increased expression level of miR-155-5p in lung tissues of iHvKp-challenged mice (Fig. 1F).

In further in vitro experiments, we confirmed the M1 polarization and inflammatory activation of macrophages by iHvKp stimulation. We found an upregulation of TNF-α and IL-6 in iHvKp group macrophages (Fig. 1G). Additionally, flow cytometry analysis showed a significant polarization of macrophages toward the M1 phenotype (CD86^+^ cells) under iHvKp stimulation (Fig. 1H). We also measured the expression of CD80 (M1 marker) and CD206 (M2 marker) at the mRNA level to further validate the results of flow cytometry (Additional file 3: Fig. S1A). These results indicate that iHvKp promotes M1 polarization and inflammatory response in macrophages.

### Isolation and characterization of macrophage-derived exosomes

**Fig. 2:**
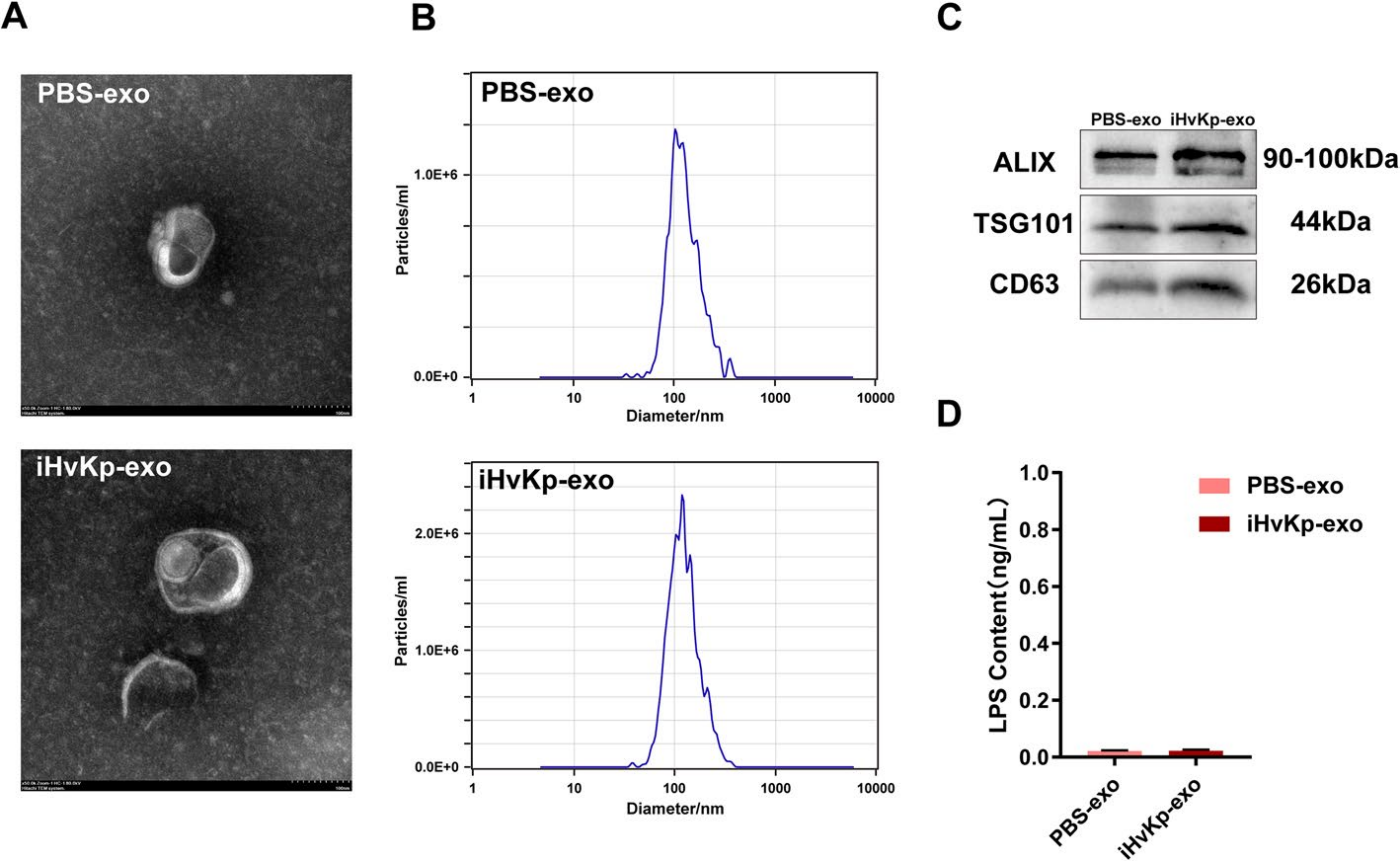
Isolation and characterization of macrophage-derived exosomes. **A** TEM image of exosomes isolated from the supernatant of iHvKp-stimulated macrophages or control group, scale bar, 100 nm. **B** NTA analysis of the isolated exosomes, including iHvKp-exo and control group. **C** Analysis of exosome markers ALIX, TSG101, and CD63 in the exosome preparation by western blot. **D** Levels of lipopolysaccharide (LPS), a bacterial cell membrane component, in the extracted exosomes, including iHvKp-exo and control group. The data are presented as mean ± SEM

To further investigate the role of exosomes derived from activated macrophages in M1 polarization and the inflammatory response of resting macrophages, we isolated macrophage-derived exosomes. TEM revealed that the exosomes exhibited cup-shaped vesicles with sizes ranging from 40 to 150 nm (Fig. 2A). To validate the size of the isolated exosomes, we conducted NTA, which confirmed that the exosomes exhibited sizes ranging from 40 to 150 nm, consistent with previously reported findings (Fig. 2B) [27]. Western blot analysis demonstrated the enrichment of exosome markers ALIX, TSG101, and CD63 in the isolated exosomes (Fig. 2C). Finally, we quantified the presence of lipopolysaccharide (LPS), a primary constituent of Gram-negative bacterial membranes, in the isolated exosomes, and the results indicated no contamination of iHvKp components in the extracted exosomes (Fig. 2D).

### IHvKp-exo induces lung injury by affecting macrophage inflammation and polarization

**Fig. 3:**
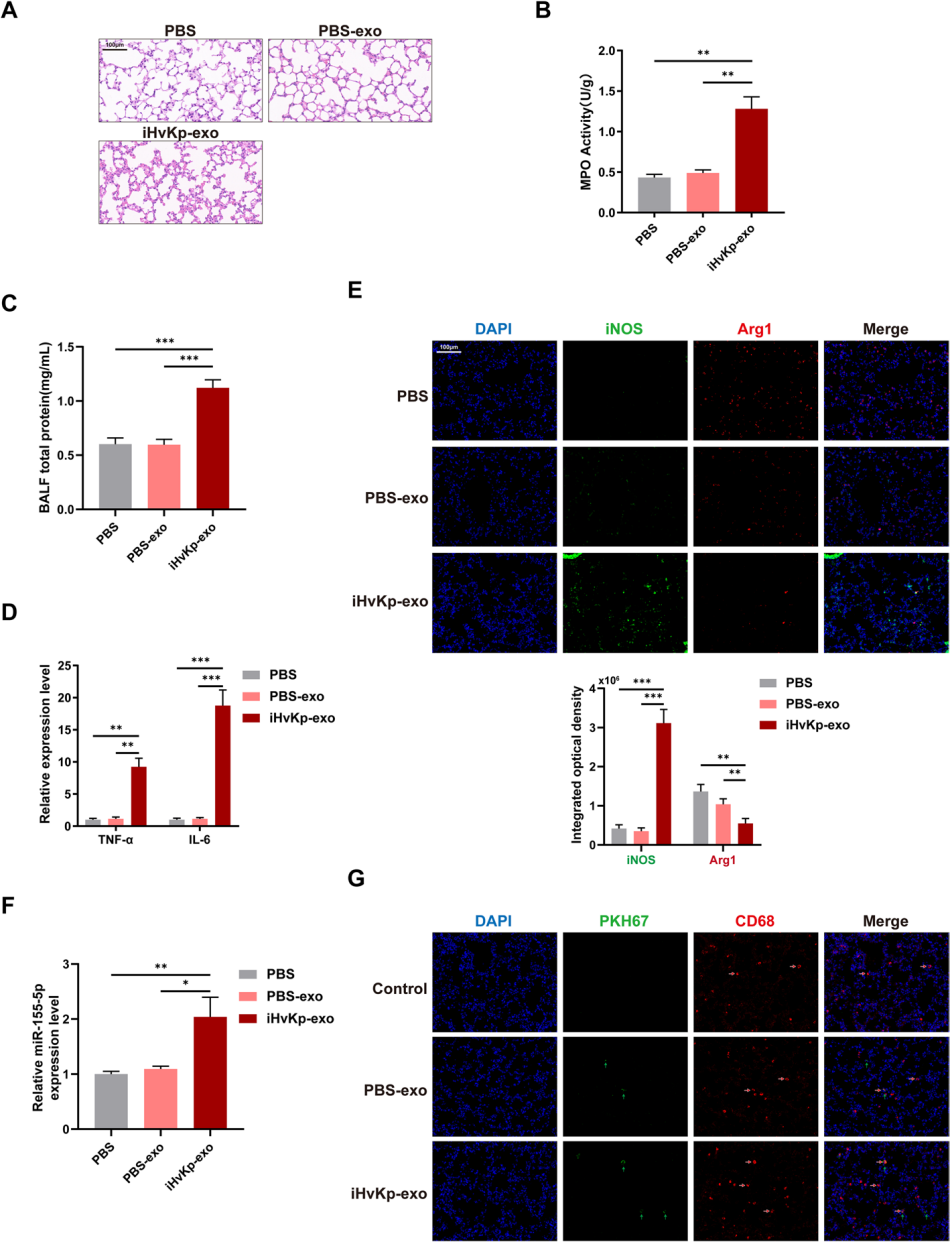
IHvKp-exo induces lung injury by affecting macrophage inflammation and polarization. **A** Lung tissue identification. The inflammatory response in lung tissues of mice injected with iHvKp-exo or control exosomes or PBS for 24 h was determined by HE staining. **B** Recruitment of inflammatory cells assessed by MPO activity. **C** Lung permeability was assessed by measuring BALF total protein concentration using a BCA assay. **D** Levels of pro-inflammatory cytokines measured by RT-PCR. **E** Immunofluorescence intensity of macrophage polarization markers in lung tissues, iNOS (green, M1) and Arg1 (red, M2). **F** MiR-155-5p expression in lung tissues measured by RT-PCR. **G** Uptake of exosomes in lung tissues. Exosomes labeled with PKH67 (green, green arrow) and macrophages labeled with CD68 (red, red arrow). Data are represented as mean ± SEM, *p < 0.05, **p < 0.01, ***p < 0.001, *n* = 3

To investigate the potential contribution of activated macrophage-derived exosomes to the development of iHvKp-induced ALI, we injected exosomes isolated from iHvKp-stimulated macrophages (iHvKp-exo) or negative control (PBS-exo) into mice. After 24 h, we collected lung tissues from the mice. HE staining revealed that mice injected with iHvKp-exo showed interstitial edema, alveolar congestion, and inflammatory cell infiltration in lung tissues, while mice injected with PBS-exo showed minimal changes (Fig. 3A). MPO assay demonstrated increased infiltration of inflammatory cells in the iHvKp-exo group (Fig. 3B). Meanwhile, there was an increase in BALF total protein in mice. (Fig. 3C). RT-PCR results showed elevated expression of inflammatory factors in the lung tissues of mice in the iHvKp-exo group (Fig. 3D), indicating that iHvKp-exo can induce inflammatory tissue damage in the mouse lungs. Immunofluorescence staining revealed increased M1 macrophage polarization in the lung tissues of the iHvKp-exo group (Figure E), suggesting that iHvKp-exo can promote inflammation and M1 macrophage polarization in the mouse lung tissues. Finally, we found the increased expression of miR-155-5p in the lung tissues of mice in the iHvKp-exo group (Fig. 3F). Additionally, we used CD68 as a specific marker to label macrophages in the tissue and observed the uptake of PKH67-labeled exosomes by macrophages using fluorescence microscopy (Fig. 3G).

### Time-dependent increase of miR-155-5p load in activated macrophage-derived exosomes

**Fig. 4:**
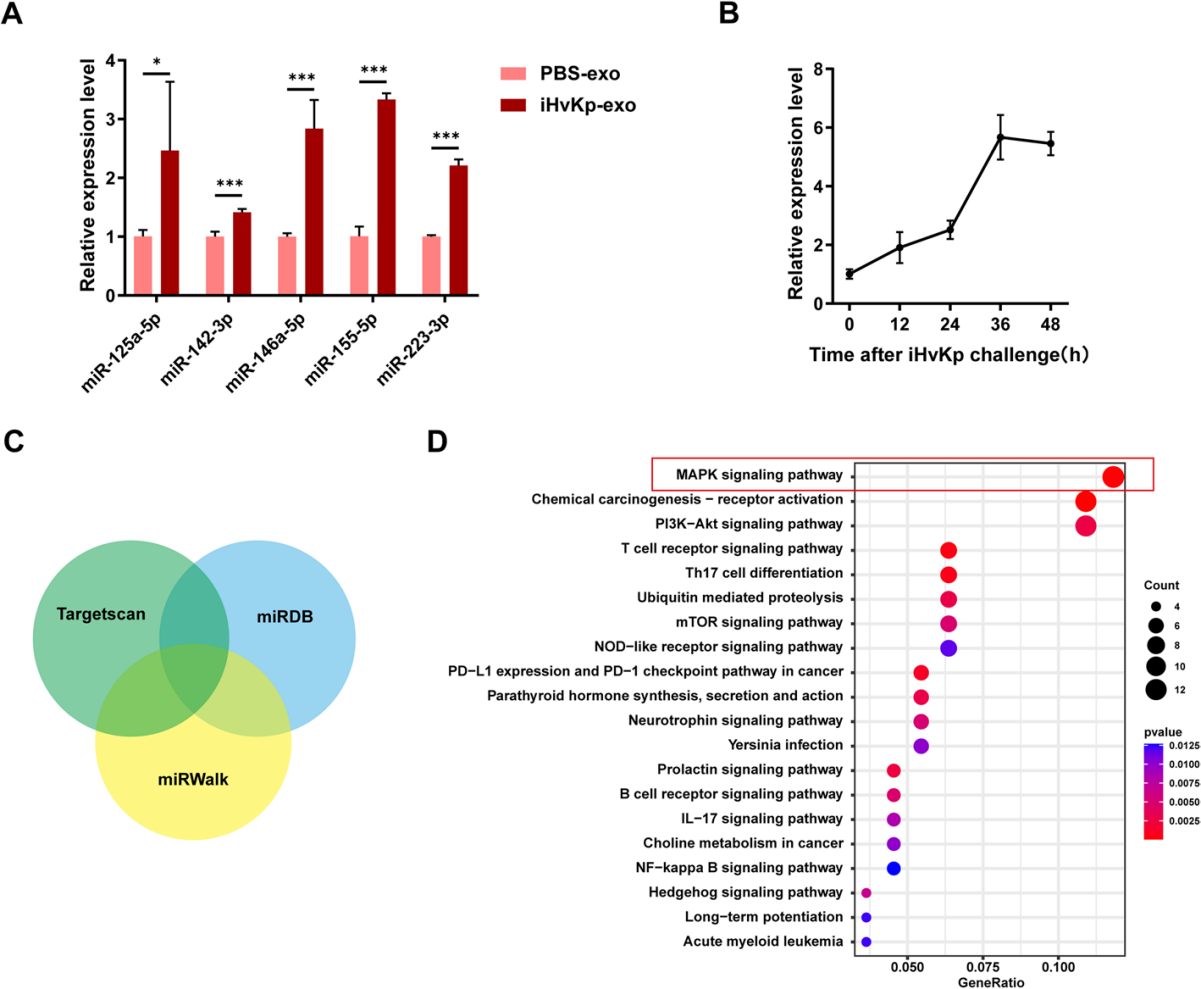
Time-dependent increase of miR-155-5p load in activated macrophage-derived exosomes. **A** Expression of inflammation-related miRNAs in iHvKp-exo and control exosomes measured by RT-PCR. **B** Expression of miR-155-5p in exosomes at different time points after iHvKp stimulation measured by RT-PCR. **C** The intersection of predicted target genes of miR-155-5p from three databases (Targetscan, miRDB, mirWalk). **D** KEGG pathway analysis was performed on potential target genes of miR-155-5p. Data are represented as mean ± SEM, *p < 0.05, **p < 0.01, ***p < 0.001, *n* = 3

To further investigate the components involved in exosomes derived from activated macrophages, we focused on miRNAs. We examined several inflammation-related miRNAs that have been reported in previous studies [28–30] and found that the levels of various inflammation-related miRNAs, including miR-155-5p, were increased in exosomes derived from iHvKp-activated macrophages compared to resting macrophages (Fig. 4A). Furthermore, we observed a time-dependent increase in the level of miR-155-5p in exosomes upon exposure to iHvKp (Fig. 4B).

To elucidate the impact and specific mechanism of exosomal miR-155-5p on macrophage inflammation and polarization, we utilized three databases (Targetscan, miRDB, mirWalk) to predict potential target genes of miR-155-5p (Fig. 4C). KEGG pathway enrichment analysis of the predicted target genes revealed that miR-155-5p's targets were mainly involved in several inflammation and proliferation-related pathways (Fig. 4D). Among them, the most enriched pathway was the Mitogen-Activated Protein Kinases (MAPK) pathway, which is widely reported to be associated with cell proliferation, differentiation, and inflammation [31, 32].

Given that extensive macrophage activation is one of the factors contributing to ALI, we proposed a hypothesis that exosomal delivery of miR-155-5p may be involved in activating resting macrophages.

### Exosomal miR-155-5p promotes macrophage M1 polarization and inflammation via activating p38^MAPK^ in vitro

**Fig. 5:**
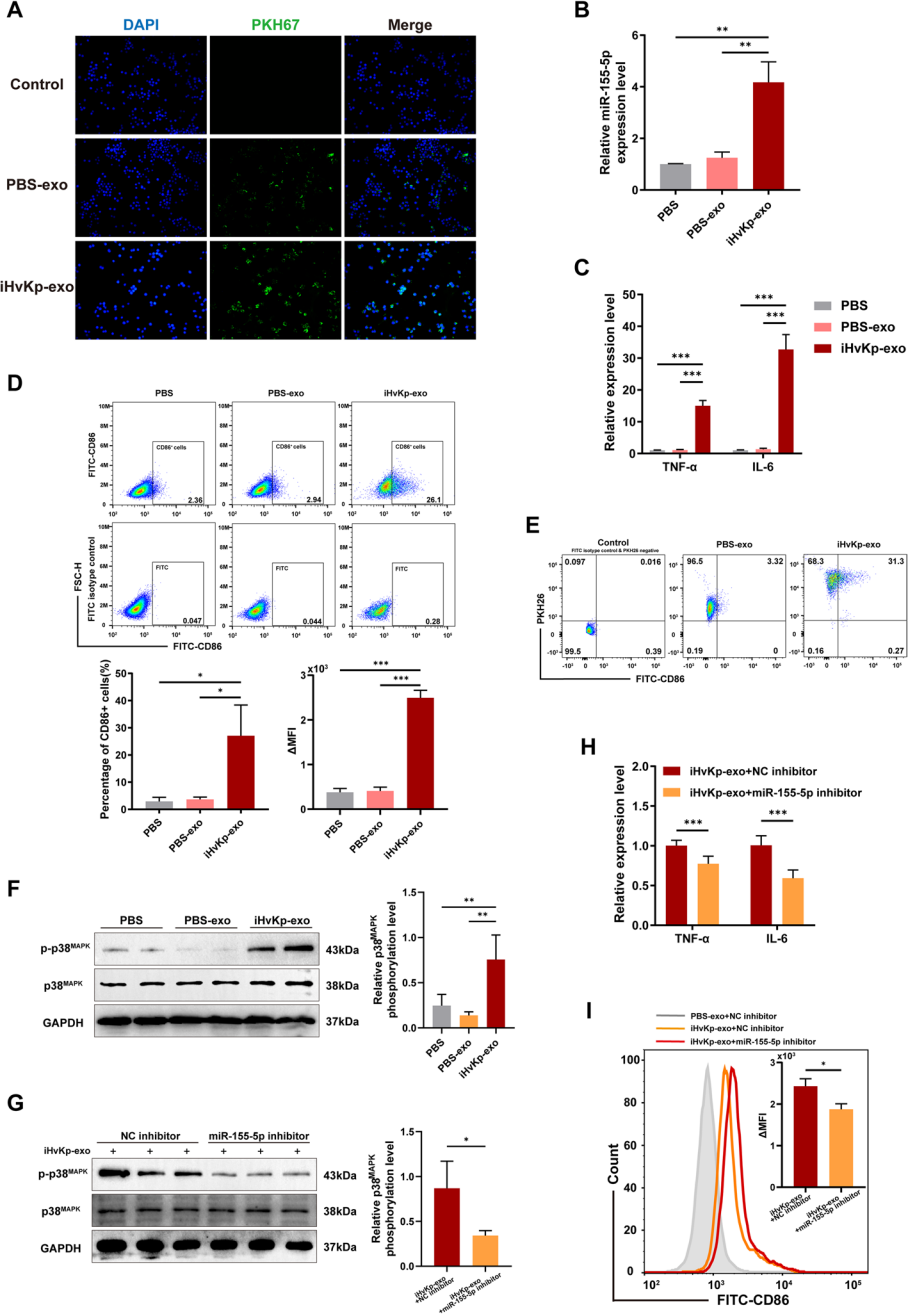
Exosomal miR-155-5p promotes macrophage M1 polarization and inflammation via activating p38^MAPK^ in vitro*.* **A** Uptake of PKH67-labeled exosomes (green) by RAW264.7 macrophages co-incubated for 6 h, observed under inverted fluorescence microscopy. **B** Expression of miR-155-5p in RAW264.7 macrophages co-incubated with iHvKp-exo or control exosomes for 48 h, measured by RT-PCR. **C** Levels of pro-inflammatory cytokines in co-incubated cells after 48 h, measured by RT-PCR. **D** Level of M1 polarization measured by flow cytometry. **E** The relationship between exosome uptake and M1 polarization of macrophages was assessed using flow cytometry. Exosomes were labeled with PKH26, while M1 macrophages were labeled with FITC-CD86 antibody. **F** Activation of p38^MAPK^ by exosomes measured by Western blotting. P-p38^MAPK^/Total p38^MAPK^ is used to assess the phosphorylation level of p38^MAPK^. **G** Activation of p38^MAPK^ in RAW264.7 macrophages transfected with miR-155-5p inhibitor or NC inhibitor before co-incubation with iHvKp-exo, evaluated by Western blotting. P-p38^MAPK^/Total p38^MAPK^ is used to assess the phosphorylation level of p38^MAPK^ **H** Pro-inflammatory cytokines expression levels measured by RT-PCR. **I** Level of M1 polarization measured by flow cytometry. Data are represented as mean ± SEM, *p < 0.05, **p < 0.01, ***p < 0.001, *n* = 3

To further validate our hypothesis, we co-incubated exosomes with macrophages directly. Under fluorescence microscopy, we observed that PKH67-labeled exosomes were taken up by macrophages (Fig. 5A). Additionally, we found a significant increase in miR-155-5p expression in macrophages treated with iHvKp-exo compared to those treated with PBS-exo (Fig. 5B). This indicates that miR-155-5p from iHvKp-exo was delivered into the recipient macrophages. Furthermore, we found a significant upregulation of inflammatory factor expression in macrophages co-incubated with iHvKp-exo (Fig. 5C). Flow cytometry analysis showed an increase of M1 macrophage polarization in the iHvKp-exo group (Fig. 5D), and the results of RT-PCR further confirmed the M1 polarization of cells in the iHvKp-exo group (Additional file 3: Fig. S1B). To further investigate the association between exosome uptake and M1 polarization of macrophages, we co-cultured PKH26-labeled exosomes with macrophages. The results showed that, regardless of the group, almost all macrophages absorbed the exosomes. Interestingly, in the iHvKp-exo group, not all macrophages that absorbed exosomes exhibited M1 polarization (Fig. 5E). In conclusion, our results demonstrate that iHvKp-exo can be absorbed by recipient macrophages, leading to their activation.

Based on the previous target gene prediction results, we observed that genes predicted by multiple databases were highly enriched in the MAPK signaling pathway, particularly the p38-MAPK signaling pathway, which has been reported to be associated with macrophage inflammation and polarization [33, 34]. Next, we observed that the iHvKp-exo treated macrophages had higher phosphorylation levels of p38^MAPK^ (Fig. 5F). Subsequently, we transfected macrophages with a miR-155-5p inhibitor before co-incubating them with exosomes. Then, we observed a decrease in the phosphorylation level of p38^MAPK^ after inhibiting miR-155-5p in macrophages (Fig. 5G). Moreover, the expression levels of inflammatory factors and the degree of M1 macrophage polarization also exhibited a certain degree of reversion after inhibiting miR-155-5p (Fig. 5H, I, Additional file 3: Figure S1C). The aforementioned results indicate that exosomal miR-155-5p activates the p38-MAPK signaling pathway in resting macrophages, leading to M1 polarization and inflammation.

### MiR-155-5p activates the p38 MAPK signaling pathway by targeting MSK1

**Fig. 6:**
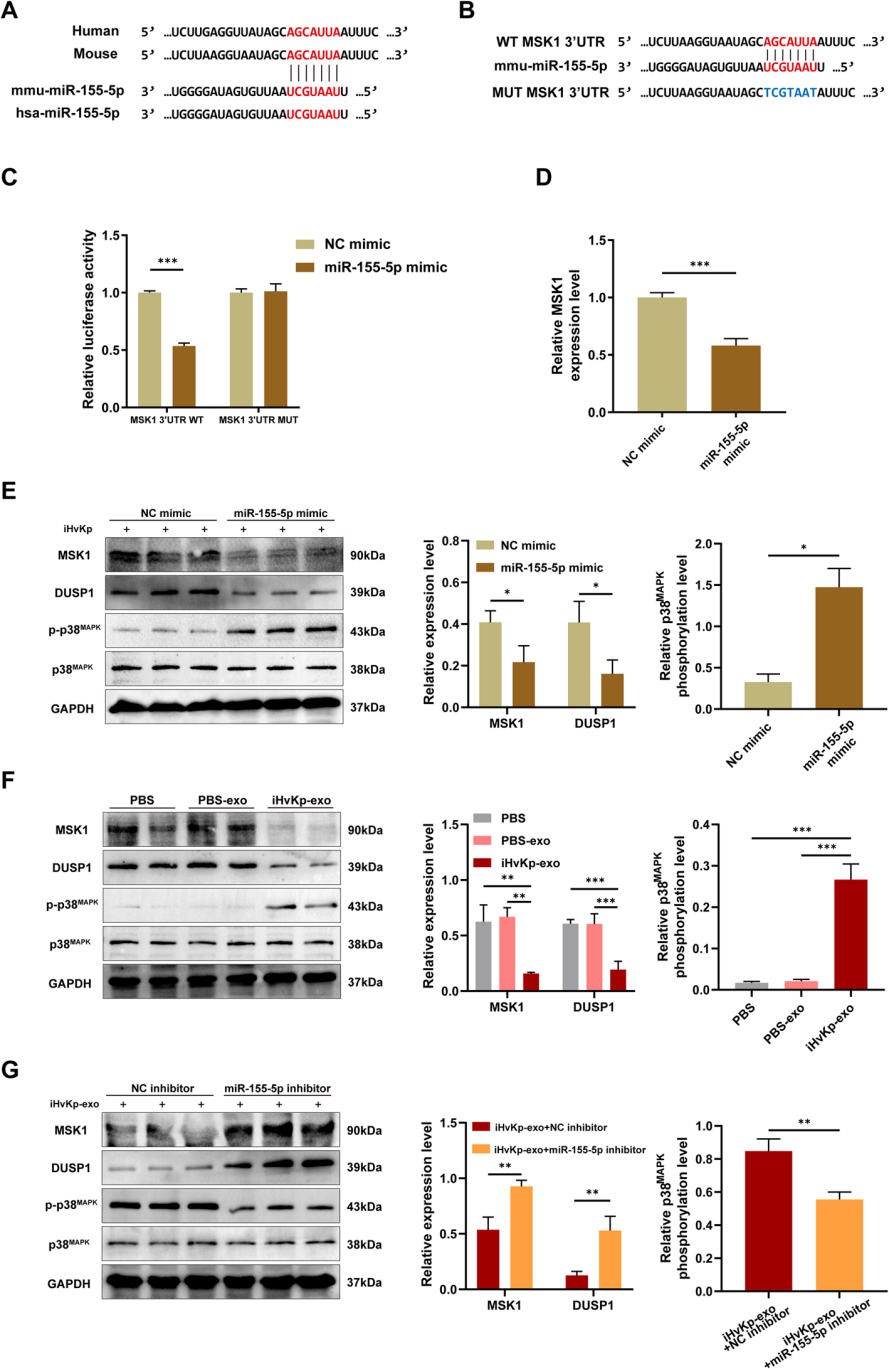
MiR-155-5p activates the p38 MAPK signaling pathway by targeting MSK1. **A** The binding target sequence of miR-155-5p on MSK1’s 3ʹ UTR exhibits conservation between humans and mice, as well as the conservation of miR-155-5p sequence between humans and mice. **B**, **D** Dual-luciferase reporter assay performed in HEK293T cells. Cells were co-transfected with dual-luciferase reporter plasmids containing the wild-type or mutant MSK1 3ʹ UTR sequence, along with NC mimic or miR-155-5p. **C** MSK1 expression was determined by RT-PCR in cells transfected with miR-155-5p mimic or NC mimic 24 h post-transfection. **E** Protein levels of MSK1, DUSP1, and p-p38^MAPK^ were determined by Western blotting. GAPDH serves as an internal reference. P-p38^MAPK^/Total p38^MAPK^ is used to assess the phosphorylation level of p38^MAPK^. **F** After co-incubation of RAW264.7 macrophages with iHvKp-derived exosomes or control exosomes for 48 h, protein expression levels of MSK1, DUSP1, and p-p38^MAPK^ were assessed by Western blotting. GAPDH serves as an internal reference. P-p38^MAPK^/Total p38^MAPK^ is used to assess the phosphorylation level of p38^MAPK^. **G** After transfection with miR-155-5p inhibitor or control for 24 h, RAW264.7 macrophages were co-incubated with iHvKp-derived exosomes, and protein expression levels of MSK1, DUSP1, and p-p38^MAPK^ were analyzed by Western blotting. GAPDH serves as an internal reference. p-p38^MAPK^/Total p38^MAPK^ is used to assess the phosphorylation level of p38^MAPK^. Data are presented as mean ± SEM. *p < 0.05, **p < 0.01, ***p < 0.001, *n* = 3

In our previous target gene prediction, we identified mitogen- and stress-activated protein kinase-1 (MSK1) as a potential target of miR-155-5p (Fig. 6A). Studies have reported that activation of MSK1 can promote M2 macrophage polarization [35]. Furthermore, studies have revealed that the knocking out MSK1 enhances macrophage responsiveness to LPS and increases their inflammatory response [36]. Dual Specificity Phosphatase 1 (DUSP1) is a known phosphorylation inhibitor of p38^MAPK^, and its expression is positively regulated by MSK1 [36, 37]. We propose that miR-155-5p targets MSK1 to downregulate its expression, subsequently reducing DUSP1 expression. This alleviates the inhibition of p38^MAPK^ phosphorylation by DUSP1 and promotes the activation of the p38 MAPK signaling pathway, thereby regulating macrophage inflammation and M1 polarization.

To validate this hypothesis, we performed a dual-luciferase reporter assay (Fig. 6B). The findings demonstrated that miR-155-5p markedly decreased the luciferase activity of the wild-type 3ʹ UTR of MSK1, while exhibiting no inhibitory effect on the mutant 3ʹ UTR of MSK1 (Fig. 6C). The mRNA detection showed a significant decrease in the expression level of MSK1 in macrophages transfected with miR-155-5p mimic (Fig. 6D). Protein level analysis also revealed downregulation of MSK1 expression in macrophages after transfection with miR-155-5p mimic (Fig. 6E). These pieces of evidence indicate that miR-155-5p inhibits MSK1 expression by directly targeting the 3ʹ UTR of MSK1 mRNA. Protein analysis also showed that the expression of DUSP1 decreased and p-p38^MAPK^ increased in the miR-155-5p mimic group, which is consistent with our hypothesis (Fig. 6E).

Furthermore, we observed macrophages co-incubated with exosomes and found that compared to the control groups, the expression of MSK1 was reduced after treated with iHvKp-exo. Along with the decrease in MSK1 expression, the expression of DUSP1 also decreased, and the phosphorylation level of p38^MAPK^ increased (Fig. 6F). After inhibiting miR-155-5p, we observed that the effects of iHvKp-exo on MSK1, DUSP1, and p-p38^MAPK^ were reversed (Fig. 6G). The above results indicate that exosomal miR-155-5p targets and inhibits MSK1 expression, reduces DUSP1 expression, increases p38-MAPK signaling pathway activation, and thereby promotes macrophage M1 polarization and inflammation.

### MiR-155-5p inhibition alleviates iHvKp-exo or iHvKp-induced lung injury

**Fig. 7:**
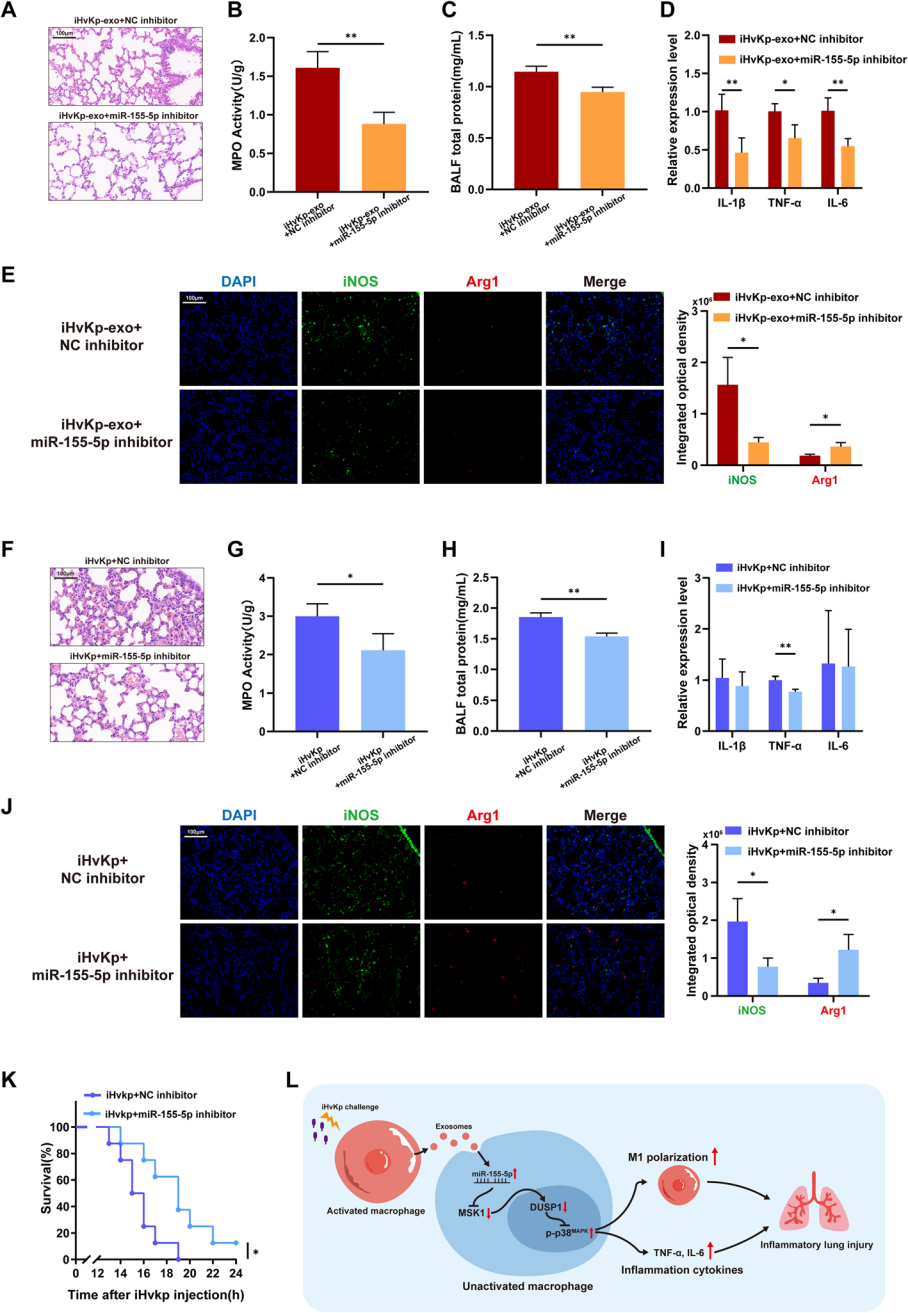
MiR-155-5p inhibition alleviates iHvKp-exo or iHvKp-induced lung injury. **A** Mice were transfected with miR-155-5p inhibitor or control (NC) 24 h before tail vein injection of iHvKp-exo (*n* = 3). After 24 h, mice were euthanized, and lung tissue inflammation was assessed by HE staining. **B** Inflammatory cell recruitment was assessed by MPO activity assay. **C** Lung permeability was assessed by measuring BALF total protein concentration using a BCA assay. **D** Levels of pro-inflammatory cytokines were determined by RT-PCR. **E** Immunofluorescence intensity of macrophage polarization markers in lung tissue, represented by iNOS (green, M1) and Arg1 (red, M2). **F** Mice were transfected with miR-155-5p inhibitor or control (NC) 24 h before tail vein injection of iHvKp (*n* = 4). Upon reaching the humane endpoint or after 24 h, mice were euthanized, and lung tissue inflammation was assessed by HE staining. **G** Inflammatory cell recruitment was assessed by MPO activity assay. **H** Lung permeability was assessed by measuring BALF total protein concentration using a BCA assay. **I** Levels of pro-inflammatory cytokines were determined by RT-PCR. **J** Immunofluorescence intensity of macrophage polarization markers in lung tissue, represented by iNOS (green, M1) and Arg1 (red, M2). **K** Survival rate analysis of mice with or without miR-155-5p inhibition after iHvKp injection (*n* = 8), was performed using the log-rank test. (**L**) Mechanism of exosomal miR-155-5p promotes macrophage M1 polarization and inflammatory response in ALI induced by HvKp infection. Data are presented as mean ± SEM, *p < 0.05, **p < 0.01, ***p < 0.001

We further explored the role of miR-155-5p in vivo. Consistent with our previous findings, we observed an upregulation of miR-155-5p expression in lung tissues following the injection of iHvKp-exo. Therefore, we transfected mice with miR-155-5p inhibitor or NC inhibitor before iHvKp-exo injection and later evaluated the inflammation and injury in lung tissues. The results showed that the transfection of miR-155-5p inhibitor alleviated interstitial edema, lung congestion, exudation, and inflammatory cell infiltration (Fig. 7A–C, Additional file 3: Figure S1D). The expression of pro-inflammatory cytokines IL-1β, TNF-α, and IL-6 also decreased (Fig. 7D). Additionally, immunofluorescence staining revealed a decrease in M1 macrophage markers and alleviation of M2 marker reduction in lung tissues (Fig. 7E). This indicates that miR-155-5p inhibitor can alleviate iHvKp-exo-induced inflammatory lung injury and macrophage activation.

Furthermore, we found an elevation of miR-155 in lung tissues of mice after injection of iHvKp. Similarly, we transfected mice with miR-155-5p inhibitor or NC inhibitor before iHvKp injection and examined the lung tissues. In mice with miR-155-5p inhibition, the lung injury caused by iHvKp, including alveolar interstitial edema, lung congestion, exudation, and inflammatory cell infiltration, was significantly reduced (Fig. 7F–H). Among the pro-inflammatory cytokines, TNF-α expression decreased, but the changes in IL-1β and IL-6 were not significant (Fig. 7I). Immunofluorescence staining showed a weakened M1 macrophage polarization in lung tissues (Fig. 7J). Importantly, we also observed that the survival time of mice with miR-155-5p inhibition was significantly longer than that of mice without inhibition after iHvKp stimulation (Fig. 7K). These findings indicate that inhibiting miR-155-5p can alleviate lung injury induced by exosomes or iHvKp, reduce inflammation and M1 macrophage polarization in the lungs, and prolong the survival time of mice with iHvKp-induced ALI.

## Discussion

The invasive and pathogenic properties of hvKp pose significant challenges in the clinical management of infections and subsequent sepsis. Sepsis is an excessive immune response triggered by bacterial infections, often driven by bacterial components such as LPS rather than intact bacteria themselves [24]. Notably, approximately 70% of severe sepsis patients exhibit negative blood cultures, further supporting the notion that sepsis is triggered by the release of bacterial components during infection [38]. In our study, to avoid the invasion and disruption caused by live bacteria, we established a sepsis model using iHvKp to better investigate ALI associated with hvKp infections.

Previous studies have demonstrated that macrophages can serve as recipient cells for exosomes derived from exogenous sources, and their cellular activities can be regulated by these exosomes [39, 40]. In sepsis, the extensive and excessive activation of macrophages may contribute to the development of ALI [10], and the role of exosomes in this process requires further investigation. Previous research has indicated that exosomes derived from peripheral circulating sources can promote macrophage proliferation and inflammatory responses, but the specific origins of these exosomes were not further explored [11]. Subsequently, it was found that neutrophil-derived exosomes activated during sepsis can induce macrophage pyroptosis and M1 polarization, providing a possible explanation for the source of these exosomes [19]. However, macrophages encounter exosomes from multiple sources during sepsis. In our study, both in vitro and in vivo, we demonstrated that exosomes derived from iHvKp-activated macrophages can be taken up by resting macrophages, leading to M1 polarization and inflammatory responses in the recipient cells. Moreover, the observed pulmonary inflammation, inflammatory cell infiltration, and tissue damage strongly suggest the significant involvement of these exosomes derived from preferentially activated macrophages in the development of ALI in the mouse model.

After being taken up by recipient cells, exosomes release their contents to regulate the biological activities of the recipient cells [15]. Recent studies have focused on the regulatory role of exosomal miRNAs in inflammatory diseases [41–43]. For instance, exosomal miR-149-3p was found to be associated with inflammatory bowel disease [41], and natural killer cells-derived exosomal miR-1249-3p could alleviate insulin resistance and inflammation in a diabetes mouse model [42]. These findings indicate that exosomal miRNAs hold promise as potential therapeutic agents or targets for intervention. Regarding exosomal miR-155-5p, recent research has discovered that inhibiting miR-155-5p targeting ETosis could alleviate mixed granulocytic asthma-related lung inflammation [44]. However, the role of exosomes in ALI associated with hvKp-induced sepsis has not been investigated. In our study, we detected multiple inflammation-related miRNAs in exosomes and found that miR-155-5p exhibited the most significant increase in exosomes derived from iHvKp-activated macrophages. Furthermore, we reported for the first time that miR-155-5p in exosomes showed an increasing trend in macrophages following bacterial exposure, indicating a selective increase in exosomal miR-155-5p secretion by macrophages upon bacterial contact. This may provide valuable insights into the occurrence and development of inflammation during sepsis.

The occurrence and progression of inflammation are often accompanied by the activation of cell inflammation-related pathways, which can lead to changes in cell proliferation, differentiation, and secretion [45, 46]. The p38-MAPK signaling pathway is a widely reported pathway associated with cancer and inflammation [33, 34]. For example, study have found a connection between the p38-MAPK pathway and proliferation and apoptosis in thyroid cancer [47]. Regarding inflammation, research has reported that a circular RNA, CircPrkcsh, participates in spinal cord injury-induced microglial cell M1 polarization through the p38-MAPK pathway [48]. Previous studies on miR-155-5p have mainly revealed its ability to promote the activation of inflammatory pathways, such as the nuclear factor κB (NF-κB) signaling pathway [49, 50]. In our study, we found that miR-155-5p in exosomes can activate the p38-MAPK signaling pathway. In light of this finding, we predicted a novel target of miR-155-5p, MSK1, through bioinformatics analysis and validated it in subsequent experiments. Previous studies have reported that DUSP1 can inhibit the phosphorylation of p38^MAPK^ and its expression is positively regulated by MSK1 [36, 37]. In our subsequent experiments, we confirmed that the miR-155-5p/MSK1/DUSP1 axis is a new pathway that activates the p38-MAPK signaling pathway, ultimately leading to macrophage M1 polarization and inflammatory responses.

Furthermore, we demonstrated the therapeutic potential of targeting miR-155-5p in ALI induced by iHvKp or iHvKp-exo. Inhibition of miR-155-5p led to reduced macrophage M1 polarization and alleviated lung inflammatory tissue damage. In an animal model of pyoseptic pneumonia-associated ALI induced by iHvKp, inhibiting miR-155-5p improved lung tissue damage and survival rate, providing a novel target for the treatment of hvKp-related ALI.

However, our study has some limitations. For example, in the exosome effector experiments, we were unable to exclude the potential impact of other contents, such as other miRNAs, which may have a minor influence on the effects of exosomes and require further investigation. Nonetheless, our experiments elucidated the pro-inflammatory effects of exosome-derived miR-155-5p and its specific mechanisms. Additionally, we also observed that not all macrophages that absorbed iHvKp-exo exhibited M1 polarization. We speculate that this may be because not all cells producing the exosomes we collected were activated by iHvKp (resulting in the exosomes we extracted not all having an activating effect on macrophages), or it could be that there is always a portion of macrophages that are less prone to activation (as shown in Fig. 1H, iHvKp stimulation did not lead to complete M1 polarization in all macrophages, and these cells were also the source of the exosomes we extracted). As for why macrophages are not uniformly activated when exposed to stimuli (exosomes or bacteria), this is an intriguing question that warrants further investigation in the future.

## Conclusions

In summary, our research provides evidence that exosomal miR-155-5p, derived from activated macrophages, is significantly upregulated in ALI induced by iHvKp. In vivo, these exosomes activate macrophages and contribute to tissue damage. We also demonstrated that these effects are mediated by miR-155-5p targeting a novel target, MSK1. Overall, our findings not only provide a novel explanation for the widespread activation of macrophages in hvKp-induced ALI but may also offer new targets and a theoretical basis for the clinical management of ALI caused by hvKp infections (Fig. 7L).

### Supplementary Information

**Additional file 1: Table S1.** Primers used for qPCR

**Additional file 2. **Additional Information about the Mouse ALI Model Induced by iHvKp.

**Additional file 3: Fig. S1.** **A** The CD80 (M1 marker) and CD206 (M2 marker) of macrophages were determined by RT-PCR after 12 h co-culture with iHvKp or PBS. **B** The CD80 (M1 marker) and CD206 (M2 marker) of macrophages were determined by RT-PCR after 24 h co-culture with iHvKp-exo, PBS-exo, or PBS. **C** Macrophages transferred NC/miR-155-5p inhibitor for 24 h. Then, The CD80 (M1 marker) and CD206 (M2 marker) of macrophages were determined by RT-PCR after 24 h co-culture with iHvKp-exo. **D **Mice were transfected with the NC inhibitor 24 h before receiving a tail vein injection of PBS (n = 3). After 24 h, the mice were euthanized, and lung tissue inflammation was assessed through HE staining (In the control group, only PBS was injected before euthanasia.) Data are presented as mean ± SEM, *p < 0.05, **p < 0.01, ***p < 0.001.

## Data Availability

The data that support the findings of this study are available from the corresponding author upon reasonable request.

## Abbreviations

hvKp: Hypervirulent *Klebsiella pneumoniae*
ALI: Acute lung injury
RT-PCR: Reverse transcription-polymerase chain reaction
miRNA: MicroRNA
MSK1: Mitogen- and stress-activated protein kinase-1
MAPK: Mitogen-activated protein kinases
TNF: Tumor necrosis factor
IL: Interleukin
CFU: Colony-forming unit
MPO: Myeloperoxidase
MOI: Multiplicity of infection
NTA: Nanoparticle tracking analysis
TEM: Transmission electron microscopy
LPS: Lipopolysaccharide
DUSP1: Dual specificity phosphatase 1
MFI: Mean fluorescence intensity
