# Supplementary material for: Exosomal miR-155-5p drives widespread macrophage M1 polarization in hypervirulent Klebsiella pneumoniae-induced acute lung injury via the MSK1/p38-MAPK axis
Source: Cell Mol Biol Lett. 2023 Nov 13;28:92. doi: 10.1186/s11658-023-00505-1 (PMC10641976; doi:10.1186/s11658-023-00505-1)
Supplement: Supplementary file 1 — Additional file 1: Table S1. Primers used for qPCR [file 11658_2023_505_MOESM1_ESM.pdf]

Additional file 1

Table S1. Primers used for qPCR

	Primer（F）	Primer（R）
CD80	TGAGTCTGGAAACCCATCTGC	TGTGGTTGCGAGTCGTATTG
CD206	GCTTCCGTCACCCTGTATGC	TCATCCGTGGTTCCATAGACC
IL-1β	GAAATGCCACCTTTTGACAGTG	TGGATGCTCTCATCAGGACAG
TNF-a	GCCGATGGGTTGTACCTTGT	TCTTGACGGCAGAGAGGAGG
IL-6	CTGCAAGAGACTTCCATCCAG	AGTGGTATAGACAGGTCTGTTGG
MSK1	GTGCTTCACCCTCCACTACG	TGAGACTGGAATGGCACCTG
GAPDH	TGGAAAGCTGTGGCGTGATG	TACTTGGCAGGTTTCTCCAGG
miR-125a-5p	TCCCTGAGACCCTTTAACCTGTGA	Supplied directly from the qPCR kit
miR-142-3p	CCGCGTGTAGTGTTTCCTACTTTATGGA	Supplied directly from the qPCR kit
miR-146a-5p	CCGTGAGAACTGAATTCCATGGGTT	Supplied directly from the qPCR kit
miR-155-5p	CCGCGTTAATGCTAATTGTGATAGGGGT	Supplied directly from the qPCR kit
miR-223-3p	CCGTGTCAGTTTGTCAAATACCCCA	Supplied directly from the qPCR kit
U6	CTCGCTTCGGCAGCACA	Supplied directly from the qPCR kit
