# Supplementary material for: Exosomal miR-155-5p drives widespread macrophage M1 polarization in hypervirulent Klebsiella pneumoniae-induced acute lung injury via the MSK1/p38-MAPK axis
Source: Cell Mol Biol Lett. 2023 Nov 13;28:92. doi: 10.1186/s11658-023-00505-1 (PMC10641976; doi:10.1186/s11658-023-00505-1)
Supplement: Supplementary file 2 — Additional file 2. Additional Information about the Mouse ALI Model Induced by iHvKp. [file 11658_2023_505_MOESM2_ESM.pdf]

Additional file 2

Additional Information about the Mouse ALI Model Induced by hvKp

1. Body temperature

	Experimental start point	Experimental endpoint
Control (PBS)	36.56±0.11	36.42±0.26
Model (iHvKp)	36.42±0.26	29.3±0.73

* In each group, n=5, and the unit is in degrees Celsius (°C). The data is presented as mean ± SEM.

2. Weight

	Experimental start point	Experimental endpoint
Control (PBS)	20.76±0.50	20.74±0.62
Model (iHvKp)	21.22±0.73	18.96±0.35

* In each group, n=5, and the unit is gram (g). The data is presented as mean ± SEM.

3. Other symptoms

Compared to the control group (PBS), the model group (iHvKp) also exhibited symptoms such as diarrhea, cessation of eating and drinking, reduced activity, and disheveled fur.
