# Supplementary material for: Exosomal miR-155-5p drives widespread macrophage M1 polarization in hypervirulent Klebsiella pneumoniae-induced acute lung injury via the MSK1/p38-MAPK axis
Source: Cell Mol Biol Lett. 2023 Nov 13;28:92. doi: 10.1186/s11658-023-00505-1 (PMC10641976; doi:10.1186/s11658-023-00505-1)
Supplement: Supplementary file 3 — Additional file 3: Fig. S1. A The CD80 (M1 marker) and CD206 (M2 marker) of macrophages were determined by RT-PCR after 12 h co-culture with iHvKp or PBS. B The CD80 (M1 marker) and CD206 (M2 marker) of macrophages were determined by RT-PCR after 24 h co-culture with iHvKp-exo, PBS-exo, or PBS. C Macrophages transferred NC/miR-155-5p inhibitor for 24 h. Then, The CD80 (M1 marker) and CD206 (M2 marker) of macrophages were determined by RT-PCR after 24 h co-culture with iHvKp-exo. D Mice were transfected with the NC inhibitor 24 h before receiving a tail vein injection of PBS (n = 3). After 24 h, the mice were euthanized, and lung tissue inflammation was assessed through HE staining (In the control group, only PBS was injected before euthanasia.) Data are presented as mean ± SEM, *p < 0.05, **p < 0.01, ***p < 0.001. [file 11658_2023_505_MOESM3_ESM.pdf]

## Additional file 3: Fig S1

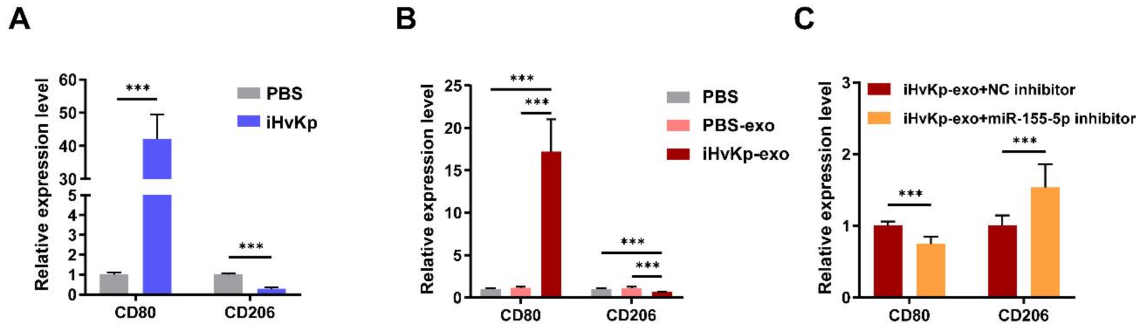


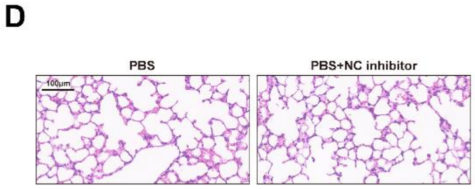


**Fig S1: A** The CD80 (M1 marker) and CD206 (M2 marker) of macrophages were determined by RT-PCR after 12 h co-culture with iHvKp or PBS. **B** The CD80 (M1 marker) and CD206 (M2 marker) of macrophages were determined by RT-PCR after 24 h co-culture with iHvKp-exo, PBS-exo, or PBS. **C** Macrophages transferred NC/miR-155-5p inhibitor for 24h. Then, The CD80 (M1 marker) and CD206 (M2 marker) of macrophages were determined by RT-PCR after 24 h co-culture with iHvKp-exo. **D** Mice were transfected with the NC inhibitor 24 hours before receiving a tail vein injection of PBS ($n$ = 3). After 24 hours, the mice were euthanized, and lung tissue inflammation was assessed through HE staining (In the control group, only PBS was injected before euthanasia.) Data are presented as mean ± SEM, $*p < 0.05$, $**p < 0.01$, $***p < 0.001$.
